# Integrated DNA walking system to characterize a broad spectrum of GMOs in food/feed matrices

**DOI:** 10.1186/s12896-015-0191-3

**Published:** 2015-08-14

**Authors:** Marie-Alice Fraiture, Philippe Herman, Loic Lefèvre, Isabel Taverniers, Marc De Loose, Dieter Deforce, Nancy H Roosens

**Affiliations:** Scientific Institute of Public Health (WIV-ISP), Platform of Biotechnology and Molecular Biology (PBB) and Biosafety and Biotechnology Unit (SBB), J. Wytsmanstraat 14, 1050 Brussels, Belgium; Institute for Agricultural and Fisheries Research (ILVO), Technology and Food Sciences Unit, Burg. Van Gansberghelaan 115 bus 1, 9820 Merelbeke, Belgium; Ghent University, Faculty of Sciences, Department of Plant Biotechnology and Bioinformatics, Technologiepark 927, 9052 Ghent, Belgium; Ghent University, Faculty of Pharmaceutical Sciences, Laboratory of Pharmaceutical Biotechnology, Ottergemsesteenweg 460, 9000 Ghent, Belgium

## Abstract

**Background:**

In order to provide a system fully integrated with qPCR screening, usually used in GMO routine analysis, as well as being able to detect, characterize and identify a broad spectrum of GMOs in food/feed matrices, two bidirectional DNA walking methods targeting p35S or tNOS, the most common transgenic elements found in GM crops, were developed. These newly developed DNA walking methods are completing the previously implemented DNA walking method targeting the t35S pCAMBIA element.

**Methods:**

Food/feed matrices containing transgenic crops (Bt rice or MON863 maize) were analysed using the integrated DNA walking system.

**Results:**

First, the newly developed DNA walking methods, anchored on the sequences used for the p35S or tNOS qPCR screening, were tested on Bt rice that contains these two transgenic elements. Second, the methods were assessed on a maize sample containing a low amount of the GM MON863 event, representing a more complex matrix in terms of genome size and sensitivity. Finally, to illustrate its applicability in GMO routine analysis by enforcement laboratories, the entire workflow of the integrated strategy, including qPCR screening to detect the potential presence of GMOs and the subsequent DNA walking methods to characterize and identify the detected GMOs, was applied on a GeMMA Scheme Proficiency Test matrix. Via the characterization of the transgene flanking region between the transgenic cassette and the plant genome as well as of a part of the transgenic cassette, the presence of GMOs was properly confirmed or infirmed in all tested samples.

**Conclusion:**

Due to their simple procedure and their short time-frame to get results, the developed DNA walking methods proposed here can be easily implemented in GMO routine analysis by the enforcement laboratories. In providing crucial information about the transgene flanking regions and/or the transgenic cassettes, this DNA walking strategy is a key molecular tool to prove the presence of GMOs in any given food/feed matrix.

**Electronic supplementary material:**

The online version of this article (doi:10.1186/s12896-015-0191-3) contains supplementary material, which is available to authorized users.

## Background

In 2014, 181.5 million hectares of genetically modified organisms (GMOs) have been planted in 28 countries [1]. On the European Union (EU) market, the commercialization of GMOs in the food/feed chain is subject to the EU legislation [2–4], which is becoming more and more complex to implement due to the increasing number and diversity of GMOs [1, 5]. The majority of EU-authorized GMOs (78.6 %) harbours the transgenic p35S element (Cauliflower mosaic virus (CaMV) 35S promoter), the transgenic tNOS element (*Agrobacterium tumefaciens* nopaline synthase terminator) or both of them, with an occurrence respectively reported of 60.7, 53.6 and 35.7 % [6–9].

To ensure the correct enforcement of the EU legislation, several GMO detection methods have been developed, mainly based on SYBR®Green and TaqMan® real-time PCR technologies. Usually, a screening is first performed with qPCR methods targeting the most common transgenic elements present in genetically modified (GM) crops (e.g. p35S and tNOS). These strategies, covering a broad spectrum of GMOs, allow to indicate the potential presence of GMOs in tested samples [6, 7, 10–13]. In case of positive responses, EU-authorized GMOs are subsequently identified and quantified using EU event-specific methods. If some observed positive screening elements, like p35S and tNOS, are not explained by these event-specific methods, the presence of EU-unauthorized GMOs can be indirectly suspected [7]. However, as most of the targeted elements originate from natural organisms (e.g. p35S from CaMV and tNOS from *Agrobacterium tumefaciens*), the confirmation of their presence can be irrefutably provided only by the characterization of the transgene flanking regions between the plant genome and the integrated cassette [7, 10, 14]. To this end, DNA walking strategies have notably been proposed in order to get this crucial information allowing to identify GM crops ([14] and references therein, [15–17] and references therein, [18–21]). However, these metods are not usually used in GMO routine analysis because they are not easily implementable by the enforcement laboratories. Recently, an integrated DNA walking strategy, better corresponding to the need of the enforcement laboratories, was developped to rapidly detect and identify EU-unauthorized GMOs, without significant additional cost and equipment [14]. This method targets the t35S element from the pCAMBIA vector, which is frequent (30 %) in transgenic plants and is absent in EU-authorized GMOs. This DNA walking approach, based on PCR, has the advantage to be fully integrated into the initial qPCR analysis as the same primers are used for the qPCR screening (detection) and the DNA walking (identification) [14, 22]. In addition, this approach was assessed as highly sensitive and able to deal with rice based mixtures and processed products, which is essential in GMO routine analysis [17].

Here, the concept of this integrated PCR-based DNA walking strategy has been adapted to also target p35S and tNOS, the most common transgenic elements found in GMOs, in order to characterize a broader spectrum of GMOs as well as to strengthen the initial DNA walking system targeting t35S from pCAMBIA. For each element, two DNA walking directions, starting from a position anchored on the sequences used for the p35S or tNOS SYBR®Green qPCR screening, have been established [6]. First, the p35S and tNOS bidirectional DNA walking methods were developed on Bt rice, as previously used for the t35S pCAMBIA method. Second, these DNA walking methods were assessed using the certified reference material (CRM) of the GM maize MON863 (9,85 %), which represents a more complex matrix due to its large genome and its low target content. Finally, in order to illustrate its applicability in routine analysis, a GeMMA Scheme Proficiency Test food matrix was submitted to the entire integrated strategy, including the qPCR screening using the p35S, tNOS and t35S pCAMBIA markers to detect the presence of GMOs and, then, the DNA walking methods, corresponding to the qPCR positive responses, allowing to characterize them.

## Results and discussion

In order to characterize a broad spectrum of both EU authorized and unauthorized GMOs, two novel DNA walking methods, based on the p35S and tNOS transgenic elements, have been developed. These methods were designed similarly to the t35S pCAMBIA DNA walking method targeting only EU unauthorized GMOs [14]. In the interest to provide an integrated approach, for each DNA walking method, the same primers allow the detection of the potential presence of GMOs containing the targeted elements (qPCR screening) as well as their characterization and identification insofar as possible (DNA walking).

### *In silico* study

Since the DNA walking approach is integrated into the screening step, the SYBR®Green primers published by Barbau-Piednoir et al., 2010 were used to target the p35S and tNOS elements [6].

As three primers are required by the DNA walking method for each targeted element, an additional primer (b) intermediate to the screening primers (a and c) was designed (Table 1). The specificity of these primers was successfully assessed *in silico*, against all EU-authorized GMOs, LLPs (Low Level Presence) and corresponding WTs (Wild-Type), using the software wEMBOSS (data not shown) [23]. Moreover, for each of the targets, two walking directions were established (p35S-F, p35S-R, tNOS-F and tNOS-R) in order to extend the GMO coverage of the integrated DNA walking strategy.

**Table 1:** Oligonucleotide primers used for the real-time PCR assays, the DNA walking approaches and the PCR confirmation of the transgenic junctions

Methods	Oligonucleotide names	Oligonucleotide sequences	Product sizes (bp)	References
SYBR®Green qPCR	p35S F	AAAGCAAGTGGATTGATGTGATA	75	[6]
	p35S R	GGGTCTTGCGAAGGATAGTG		[6]
SYBR®Green qPCR	tNOS F	GATTAGAGTCCCGCAATTATACATTTAA	69	[6]
	tNOS R	TTATCCTAGKTTGCGCGCTATATTT		[6]
SYBR®Green qPCR	t35S pCAMBIA c-F	CGGGGGATCTGGATTTTAGTA	137	[14]
	t35S pCAMBIA a-R	AGGGTTCCTATAGGGTTTCGCTC		[14]
DNA Walking	p35S-F a (p35S R)	GGGTCTTGCGAAGGATAGTG		[6]
	p35S-F b	TGTGCGTCATCCCTTACGTCAGT	/	This study
	p35S-F c	TATCACATCAATCCACTTGCTTT		[6]
DNA Walking	p35S-R a (p35S F)	AAAGCAAGTGGATTGATGTGATA		[6]
	p35S-R b	ACTGACGTAAGGGATGACGCACA	/	This study
	p35S-R c	CACTATCCTTCGCAAGACCC		[6]
DNA Walking	tNOS-F a (tNOS F)	GATTAGAGTCCCGCAATTATACATTTAA		[6]
	tNOS-F b	TTAATACGCGATAGAAAACAAAAT	/	This study
	tNOS-F c	AAATATAGCGCGCAAMCTAGGATAA		[6]
DNA Walking	tNOS-R a (tNOS R)	TTATCCTAGKTTGCGCGCTATATTT		[6]
	tNOS-R b	ATTTTGTTTTCTATCGCGTATTAA	/	This study
	tNOS-R c	TTAAATGTATAATTGCGGGACTCTAATC		[6]
PCR junction	Rice chromosome II	CCCCTAATTTCTCACAGGCC	848	This study
	tNOS-F c	AAATATAGCGCGCAAMCTAGGATAA		[6]
PCR junction	Rice chromosome III	AGGTACTCAAGCCTTTTCCAGC	1105	This study
	tNOS-F c	AAATATAGCGCGCAAMCTAGGATAA		[6]

### Development of the DNA walking methods

#### Assessment of p35S DNA walking methods

**Fig. 1:**
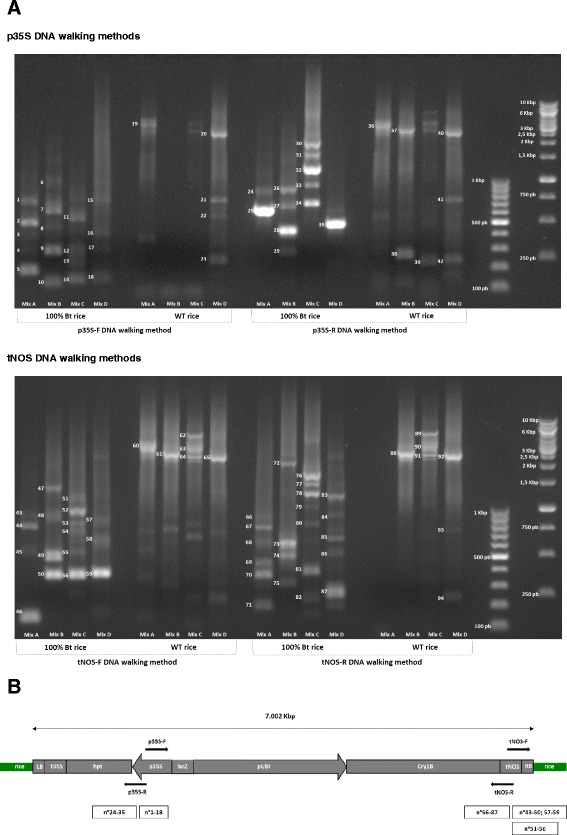
Development of the bidirectional p35S and tNOS DNA walking methods on 100 % Bt rice. **a** Visualisation of the obtained amplicons, numeroted from 1 to 94, using the p35S and tNOS DNA walking methods applied on 100 ng of 100 % Bt rice and WT rice. For each method, four different DRT primer mixes (A-D) have been used. **b** For each DNA walking method, a schematic representation of the potential start position and direction, applied on the transgenic cassette of the Bt rice, is illustatred by the black arrows. Below the transgenic cassette, the sequence covering of the obtained amplicons from the 100 % Bt rice is schematically represented by rectangles. The corresponding amplicon numbering is indicated in the Fig. 1a. LB (left border); t35S (CaMV 35S terminator); hpt (hygromycin phosphotransferase gene); p35S (CaMV 35S promoter); lacZ (LacZ alpha fragment); pUBI (maize ubiquitin promoter); Cry1B (synthetic Cry1B gene); tNOS (Agrobacterium tumefaciens nopaline synthase terminator); RB (right border); rice (rice genome) [Schema adapted from 24]

For the p35S approach, several amplicons were observed from 100 % Bt rice, corresponding to 200 000 HGEs (Haploid Genome Equivalent), for the four different degenerated random tagging (DRT) primers (A-D), including 18 amplicons for the p35S-F DNA walking method (amplicons n° 1 to 18) and 12 amplicons for the p35S-R DNA walking method (amplicons n° 24 to 35) (Fig. 1a). The size range of these amplicons was approximately from 100 bp to 1 Kbp and from 250 pb to 2 Kbp for the p35S-F and p35S-R DNA walking method, respectively. All these amplicons were consecutively analysed by sequencing to evaluate the specificity of the methods (Additional file 1).

All these characterized sequences corresponded specifically to the position of the p35S element in the transgenic cassette (Fig. 1b) [24]. As expected, these sequences present the continuity of the p35S element [GenBank:AF234296] for the p35S-F DNA walking method (amplicons n °1 to 18) and the p35S promoter [GenBank:AF234296] regulating the hygromycin resistance gene (hpt) [GenBank:AAF65337] for the p35S-R DNA walking method (amplicons n° 24 to 35) (Fig. 1b and Additional file 1).

For the WT rice sample, few amplicons (amplicons n° 19 to 23 for the p35S-F method and amplicons n° 36 to 42 for the p35S-R method) were observed (Fig. 1a) and identified as corresponding to the rice genome (Additional file 2). They are probably due to the use of DRT primers which can potentially generate a background of aspecific products, especially in absence or in low amounts of targeted sequences [25].

#### Assessment of tNOS DNA walking methods

The use of the tNOS DNA walking approach with the two walking directions using the four different DRT primers (A-D) on the 100 % Bt rice sample produces several amplicons, including 17 amplicons for the tNOS-F DNA walking method (amplicons n° 43 to 59) and 22 amplicons for the tNOS-R DNA walking method (amplicons n° 66 to 87) (Fig. 1a). The tNOS-F and tNOS-R DNA walking methods gave respectively amplicons with a size range of approximately 100 bp to 1.5 Kbp and 200 pb to 2 Kbp. To assess the specificity of the methods, all these PCR products were examined by sequencing (Additional file 1).

On the one hand, as expected, regarding the tNOS element localisation in the transgenic cassette, 100 % of the analysed amplicons coming from the tNOS-F DNA walking method have allowed to characterize the transgene flanking regions between the rice genome and the right border of the integrated pCAMBIA cassette via the amplicon sequences containing both the tNOS element and the rice genome (Fig. 1b and Additional file 1). None of the obtained amplicons presented an unexpected sequence. As the Bt rice presents two transgenic insertions, two types of transgene flanking regions were characterized: one localised between the transgenic cassette [GenBank:AY836546.1] and a genomic sequence from chromosome II of *Oryza sativa* japonica Group [GenBank:OSJNBa0016G10] identified using the amplicons generated by the DRT C primers (amplicons n° 51 to 56) and one situated between the pCAMBIA cassette [GenBank:AY836546.1] and a genomic sequence from chromosome III of *Oryza sativa* japonica Group [GenBank:OSJNBb0111B07] identified using the amplification coming from the DRT A, B and D primers (amplicons n° 43 to 50 and n° 57 to 59) [14, 17]. These results yet clearly demonstrate the importance to use four different DRT primer mixes. Indeed, the difference in affinity of these DRT primers allows increasing the likelihood to successfully characterize all targets [14, 17]. In addition, the right border of the pCAMBIA cassette on chromosome II was shorter of two base-pairs compared to the one on chromosome III (Additional file 1). These two transgene flanking regions were also properly confirmed by sequencing of PCR products obtained in using primers annealing to the pCAMBIA cassette and chromosome II or III (Table 1; Additional file 3).

On the other hand, as expected, all PCR products generated from the tNOS-R DNA walking method allow to characterize the continuity of the tNOS element (amplicons n° 71, 82 and 87) as well as, for the longer ones, the flanking region between the tNOS element [GenBank:HQ593861.1] and the Cry1B gene [GenBank:KC414884.1] conferring an insect resistance (amplicons n° 66 to 70, n° 72 to 81 and n° 83 to 86) (Fig. 1b and Additional file 1). 100 % of the analysed amplicons corresponded to the expected sequences.

Similarly to the p35S DNA walking methods, the bidirectional tNOS approach presents uniquely specific amplifications further to the analysis of the Bt rice sample while few aspecific amplicons (Fig. 1b), corresponding to the rice genome, were generated from the WT rice material (amplicons n° 60 to 65 for the tNOS-F method and amplicons n° 88 to 94 for the tNOS-R method) (Additional file 2).

### Practical application of the DNA walking methods

#### Analysis of GM maize

To test the developed p35S and tNOS bidirectional DNA walking methods on a more complex food matrix than rice in term of genome size and target amount, GM maize MON863 9.85 % (ERM-BF416c), corresponding to 3 788 HGEs, was selected as it possesses both the p35S and tNOS elements in its transgenic cassette [6, 26].

**Fig. 2:**
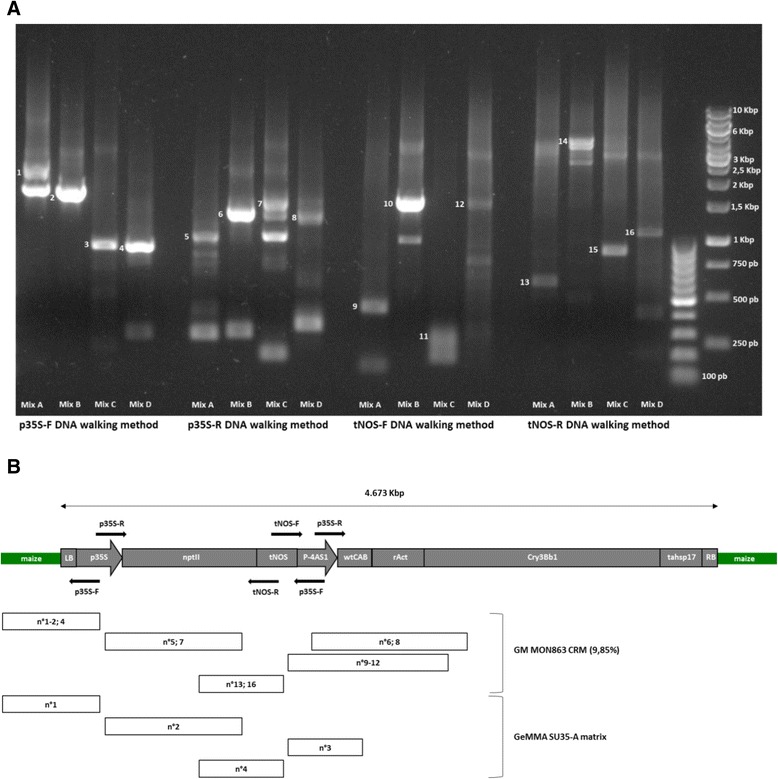
Application of the bidirectional p35S and tNOS DNA walking methods on GM maize matrices. **a** Visualisation of the obtained amplicons using the p35S and tNOS DNA walking methods applied on 100 ng of the GM MON863 maize CRM (9.85 %). For each method, four different DRT primer mixes (A-D) have been used. The analyzed amplicons are indicated by a numerotation going from 1 to 16. **b** For each DNA walking method, a schematic representation of the potential start position and direction, applied on the transgenic cassette of the GM maize MON863, is illustatred by the black arrows. Below the transgenic cassette, the sequence covering of the selected amplicons from the GM MON863 maize CRM (9.85 %) and the GeMMA proficiency test food matrix (GeMMA SU35-A) is schematically represented by rectangles. The corresponding amplicon numbering is indicated in the Fig. 2a and Additional file 6. LB (left border); p35S (CaMV 35S promoter); nptII (neomycin phosphotransferase II gene); tNOS (Agrobacterium tumefaciens nopaline synthase terminator); p4-AS1 (modified CaMV 35S promoter); wtCAB (Wheat major chlorophyll a/b binding protein gene); rAct (Rice Actin intron); Cry3Bb1 (synthetic Cry3Bb1 gene); tahsp17 (Wheat heat shock protein terminator); RB (right border); maize (maize genome) [Schema adapted from 27 and 30]

First, the presence of these elements in the tested CRM sample was confirmed by SYBR®Green qPCR screening (Additional file 4). Then, several amplicons were generated by each DNA walking method with a size ranging from approximately 200 bp to 4 Kbp (Fig. 2a). In order to obtain the most informative sequences, the amplicon with the highest size for each DRT primer mix in each applied DNA walking method was selected to be sequenced (Additional file 5).

Most of the selected amplicons from the p35S-F DNA walking method present the 5’ transgene flanking region between the maize genome [GenBank:DQ490951.2] and the p35S promoter [GenBank:KJ608136.1] from the transgenic cassette of MON863, as previously published (Fig. 2b and Additional file 5) [27, 28]. This transgene flanking region, confirming the presence of GM maize MON863, is also targeted by the EU event-specific qPCR method to identify and quantify this GMO [29]. Only one tested amplicon (n °3) showed an aspecific sequence corresponding to the WT maize genome [GenBank:AC196084; Zea mays BAC clone CH201-52A17 from chromosome 5] (Fig. 2a and Additional file 2). A possible explanation is that the tested sample contains primarily WT maize material and only a relative low amount of the target.

For the p35S-R DNA walking method, two different types of sequences were observed due to the presence of two p35S promoters in the transgenic cassette of GM MON863 maize (Fig. 2b) [27, 30]. On the one hand, the continuity of the p35S promoter [GenBank:KJ608136.1; Zea mays transgenic line MON863 promoter region] regulated the neomycin phosphotransferase gene (nptII) from *A. tumefaciens* [GenBank:AAF65400.1] which confers a resistance to kanamycin (Fig. 2b and Additional file 5). On the other hand, a part of the p35S promoter [GenBank:JX139718.1], followed by the 5’ upstream sequence of the Wheat major chlorophyll a/b binding protein gene (wtCAB) [GenBank:X05823.1] and by the Rice Actin intron (rAct) [GenBank:EU155408.1; X63830.1], regulated the synthetic Cry3Bb1 gene [GenBank:CS409981.1; GX181970.1], providing insect resistance (Fig. 2b and Additional file 5). The information acquired from these both types of sequences allows a better characterization of the transgenic cassette.

Using the tNOS-F DNA walking method, all analysed amplicons showed a part of the tNOS terminator from *A. tumefaciens* [GenBank:JN400387.1] followed by a modified p35S promoter, including in upstream of four repeats of a short activating sequence (21pb), referred to as 4-AS1 promoter [GenBank:JX139718.1] [27, 28, 30]. This modified p35S promoter was then followed by the 5’ upstream sequence of the Wheat major chlorophyll a/b binding protein gene (wtCAB) [GenBank:X05823.1] and the Rice Actin intron (rAct) [GenBank:EU155408.1; X63830.1] to regulate the Cry3Bb1 gene [GenBank:CS409981.1; GX181970.1] (Fig. 2b and Additional file 5).

The tNOS-R DNA walking method provided the sequence of the nptII selection marker [GenBank:AAF65400.1] followed by the tNOS terminator [GenBank:JN400387.1] (Fig. 2b and Additional file 5). An aspecific sequence corresponding to the WT maize genome [GenBank:AC196084], identical to the amplicon n °3 from the p35S-F DNA walking method, was observed for the amplicons n° 14 and 15 (Additional file 2).

Most of the tested amplicons (81.25 %) derived from all the DNA walking methods presented a sequence corresponding to the GM targets. Based on these data, the presence of GM MON863 maize in the tested sample was clearly identified by isolation and sequencing of its junction between the maize genome and the transgenic cassette. In addition, this strategy allows to reconstruct 2.727 Kbp of the integrated transgenic cassette, going from the left border to a part of the gene Cry3Bb1, in agreement with the published information (Fig. 2b and Additional file 5) [27, 28, 30]. These results also highlight that the proposed DNA walking strategy is able to identify GMOs from different plant species.

#### Analysis of the food matrix

In order to illustrate its applicability in GMO routine analysis by the enforcement laboratories, the entire workflow of the integrated system was applied on a food matrix (GeM SU34-A) from a GeMMA Scheme Proficiency Test containing 1.2 % of GM maize MON863 event, corresponding to 461 HGEs.

First, similarly to the GMO routine analysis, the GeMMA food matrix was submitted to the SYBR®Green qPCR screening using the p35S, tNOS and t35S pCAMBIA screening markers allowing to detect the potential presence of GMOs (Table 1). As expected, a positive signal was observed for the p35S and tNOS screening markers while the t35S pCAMBIA screening marker gave a negative signal (Additional file 4), suggesting the potential presence of GMOs in the tested food matrix.

Second, based on the positive signals obtained from the screening qPCR analysis, the bidirectional p35S and tNOS DNA walking approaches were selected to be applied on the sample. In doing so, the potential presence of GMOs will be confirmed by the characterization of their sequences.

All applied DNA walking methods were able to produce amplicons in a size range from approximately 200 bp to 1.5 Kbp (Additional file 6). In order to follow an efficient workflow suitable for GMO routine analysis, only one amplicon, chose for its large size as well as for its ease to be selected on an electrophoresis gel, was sequenced for each DNA walking method (Additional file 6).

With all these DNA walking methods, 100 % of the analysed amplicons presented sequences specific to the GM target. Indeed, when using p35S-F DNA walking, the transgene flanking region between the maize genome [GenBank:DQ490951.2] and the p35S promoter from the transgenic cassette of MON863 [GenBank:KJ608136.1] was identified, proving the presence of this GMO in the tested sample [27, 28]. The p35S-R DNA walking method presented the continuity of the p35S promoter [GenBank:KJ608136.1] regulating the nptII selection marker [GenBank:AAF65400.1]. From the tNOS-F DNA walking method, a part of the tNOS terminator [GenBank:JN400387.1], followed respectively by the 4AS-1 promoter [GenBank:JX139718.1] and the Wheat major chlorophyll a/b binding protein gene (wtCAB) [GenBank:X05823.1], was detected. Via the tNOS-R DNA walking method, the sequence of the nptII gene [GenBank:AAF65400.1] regulated by the tNOS terminator [GenBank:JN400387.1] was observed (Fig. 2b and Additional file 5 and 6).

All these sequences indubitably prove the presence of the GM MON863 maize event in the GeMMA food matrix sample though the identification of its junction between the maize genome and the transgenic cassette as well as the partial reconstruction of its transgenic cassette, in agreement with the published information [27, 28, 30].

Similarly to the t35S pCAMBIA DNA walking method, the good specificity of the newly developed DNA walking methods (p35S-F, p35S-R, tNOS-F, tNOS-R) was illustrated in this study since almost all of the sequences from the analysed amplicons generated from the Bt rice (100 %), MON863-9.85 % (81.25 %) and Gemma proficiency test (100 %) matrices corresponded to the GM targets [14, 17]. The success of this strategy is mainly due to the specificity of the target-specific primers, allowing to initially amplify the targets by PCR and, then, to enrich them by two successive semi-nested PCRs (Table 1).

## Conclusion

In order to provide an integrated system able to detect, characterize and identify a broad spectrum of both EU authorized and unauthorized GMOs in food/feed matrices, two bidirectional DNA walking methods targeting p35S or tNOS, the most common transgenic elements, were developed to be anchored on the sequences used for the p35S or tNOS qPCR SYBR®Green screening described by Barbau-Piednoir et al., 2010 [6]. These DNA walking methods also allow to strengthen the previously published t35S pCAMBIA DNA walking method in order to currently target around 75 % of the GM crops [14, 17], personal communication.

First, the p35S and tNOS bidirectional DNA walking methods were developed and assessed for their specificity using 100 % Bt rice. These methods were evaluated as highly specific since no aspecific amplifications were generated in presence of the target. Second, the developed DNA walking methods were tested on a more complex maize food matrix, in term of genome size, containing approximately 10 % of the GM maize MON863 event. Finally, the entire workflow of the integrated system, including the detection of the potential presence of GMOs by qPCR screening with the p35S, tNOS and t35S pCAMBIA markers and, subsequently, the confirmation of their presence using the DNA walking methods corresponding to the previously obtained qPCR responses, was applied on a GeMMA Scheme Proficiency Test matrix, containing 1.2 % of the GM maize MON863 event, to illustrate its applicability in GMO routine analysis by the enforcement laboratories. For all tested matrices, the p35S and the tNOS bidirectional DNA walking methods were successfully applied as the GMO presence was proven via the characterization of the junction between the transgenic cassette and the plant genome as well as of a part of the transgenic cassette.

In addition to its clear benefit in GMO detection, this integrated system has the advantage to present a simple procedure and a short time-frame to get the results. However, in order to analyse even more easily the PCR products derived from the DNA walking methods, some adaptations in the entire DNA walking workflow could be done regarding the purification of the generated amplicons excised from the electrophoresis gel and the subsequent sequencing using Sanger technology. Indeed, even if the initial DNA walking workflow remains simple, in case of matrices containing several GMOs, harbouring the same targeted element, the purification of the potential numerous amplicons excised from the electrophoresis gel and the subsequent Sanger sequencing could be cumbersome. This situation could be for instance encountered with matrices presenting a low amount of EU-unauthorized GMOs mixed with EU-authorized GMOs harbouring the elements p35S and/or tNOS, very frequently observed in GM crops. In this scenario, the obtained amplicons will present different sequences, representing potentially one GMO per observed DNA fragment. Therefore, the simplified workflow, consisting in selecting the largest size amplicons to obtain the most informative sequences, does not guarantee the entire representativeness of GMOs present in the tested sample. Consequently, it’s preferable to analyse all amplicons observed on the electrophoresis gel and to eventually them using Sanger technology, which may be a quite laborious work. In the future, this difficulty could be circumvented in replacing the step related to the purification of the amplicons excised from the electrophoresis gel and the subsequent Sanger sequencing by a high-throughput Next-Generation-Sequencing approach, as suggested by Liang et al., 2014 [18].

## Methods

### Plant material

Grains of an insect resistant transgenic Bt rice (*Oryza sativa L. Japonica cv Ariete*), transformed by *Agrobacterium tumefaciens* with the binary vector pCAMBIA 1300 containing the synthetic Cry1B gene from *Bacillus thuringiensis*, and its corresponding wild-type (WT) were used in this study [24]. The CRM of the GM maize MON863 9.85 % (ERM-BF416c) in the form of seed powder was obtained from the Institute for Reference Materials and Measurements (IRMM, Geel, Belgium). The food matrix (GeM SU34-A), coming from a GeMMA Scheme Proficiency Test, is a maize flour, tumble blended for 50 h, containing 1.2 % (w/w) of 100 % GM maize MON863.

### DNA extraction, concentration and purity

Using a CTAB-based procedure (ISO 21571) in combination with the Genomic-tip20/G (QIAGEN, Hilden, Germany), DNA was extracted from a homogenous powder of rice grain obtained by manual grinding. Adapted from the EU-RL GMFF validated method, this DNA extraction method was carried out by four main successive steps: Extraction of proteins, polysaccharides and organic components, precipitation of DNA in the presence of C-hexadecyl-Trimethyl-Ammonium-Bromide (CTAB), purification of DNA using a tip20 column and precipitation of DNA with isopropanol [31, 32]. DNA concentration was measured by spectrophotometry using the Nanodrop® 2000 (ThermoFisher, DE, USA) device and the DNA purity was evaluated using the A260/A280 and A260/A230 ratios. DNA extraction, concentration and purity of the CRM and the food matrix (GeM SU34-A) were carried out as previously described [33].

### qPCR SYBR®Green technology

All qPCR assays were performed as described in Barbau-Piednoir et al., 2010 and Fraiture et al., 2014 using the primers indicated in Table 1 [6, 14]. More precisely, a standard 25 μl reaction volume was applied containing 1X SYBR®Green PCR Mastermix (Diagenode, Liège, Belgium), 250 nM of each primer and 5 μl of DNA (10 ng/μl). The qPCR cycling program consisted of a single cycle of DNA polymerase activation for 10 min at 95 °C followed by 40 amplification cycles of 15 s at 95 °C (denaturing step) and 1 min at 60 °C (annealing-extension step). The program for melting curve analysis was performed by gradually increasing the temperature from 60 to 95 °C in 20 min (±0.6 °/20 s). All runs were performed on an iQ™5 real-time PCR detection system (BioRad, Hemel Hempstead, UK). For each assay, a “No Template Control” (NTC) was included.

### DNA walking approach

#### Development and assessment of oligonucleotide primers

Two DNA walking approaches have been developed to target the p35S or tNOS elements. For each method, three target-specific primers are required to carry out first the DNA walking (a) and then the first (b) and the second (c) semi-nested PCR rounds. To provide an integrated approach, the design of the target-specific primers a and c is based on the sequences from the SYBR®Green real-time PCR screening markers p35S or tNOS published by Barbau-Piednoir et al., 2010 [6]. An intermediate primer, corresponding to the target-specific primer b, was additionally designed. From each targeted transgenic element, two walking directions, called forward (F) and reverse (R) methods, have been performed (Fig. 1b; Table 1). Using the program “wprimersearch” from the software “wEMBOSS”, that mimics PCR amplification, the specificity of oligonucleotide primers was initially assessed *in silico* [23].

#### DNA walking strategy

##### DNA walking and double semi-nested PCR reactions

The DNA walking strategy previously described by Fraiture et al., 2014 was adapted in this study to target the transgenic element p35S or tNOS [14]. Similarly, a first reverse target-specific primer (a) and one kind of the degenerated random tagging primer (DRT) mix (A-D) were applied, in a first step, followed by two semi-nested PCR rounds using target-specific primers (b and c), that are each time nested to the previous reverse target-specific primer, combined to universal tagging primers (UAP-N1 and UAP-N2) [14]. All these methods were applied on 100 ng of DNA from 100 % of Bt rice and its corresponding WT as well as on 100 ng of DNA from the food matrix (GeM SPU34-A) and its corresponding CRM (GM maize MON863 9.85 %). Moreover, a NTC was included for each assay. PCR mixes and conditions were carried out according to the manufacturer’s instructions (APAgene™ GOLD Genome Walking Kit from BIO S&T, Montréal, Canada). The final PCR products were analysed by electrophoresis on a 1 % agarose gel (INVITROGEN, CA, USA) (100 V, 400 mA, 60 min) in view to further analysis allowing to identify the generated sequences.

##### Analysis and workflow

In order to assess the specificity of the developed p35S and tNOS bidirectional methods, all the visualized amplicons produced from the 100 % Bt rice and WT rice were excised from agarose gel and purified using the Wizard® SV Gel and PCR Clean-Up System (Promega, WI, USA) to then be sequenced and identified.

Next, to test the developed methods on a maize matrix, the CRM of maize MON863 (9,85 %) was used and a workflow convenient for the GMO routine analysis was followed. For each DNA walking method, only the longest and easily selectable amplicon observed for each DRT primer mix was excised from agarose gel and purified using the Wizard® SV Gel and PCR Clean-Up System (Promega, WI, USA) to be sequenced.

To test the applicability of the entire integrated system, a simplified workflow was used for the food matrix (GeM SPU34-A). Only the longest and easily selectable amplicon observed for each DNA walking method was excised from agarose gel and purified using the Wizard® SV Gel and PCR Clean-Up System (Promega, WI, USA) to be sequenced.

### Cloning and sequencing

Three different sequencing approaches were used to obtain the sequence of the selected amplicons. First, a direct sequencing was applied using the corresponding target-specific c primer or the UAP-N2 primer. Second, in case of an unsatisfying size or quality of the obtained sequences, two other sequencing approaches were carried out. On the one hand, a cloning strategy was performed. The amplicons were cloned into the pGEM®-T Easy Vector Systems (PROMEGA, WI, USA), according to the manufacturer’s instructions. A PCR was carried out on colonies using pGEM®-T Easy Vector primers (T7: TAATACGACTCACTATAGGG; SP6: ATTTAGGTGACACTATAGAAT) and was analyzed by electrophoresis on a 1 % agarose gel (INVITROGEN, CA, USA) (100 V, 400 mA, 60 min). The colonies presenting a fragment of the correct size were then sequenced [34]. On the other hand, an “enrichment” strategy, based on a PCR amplification using the corresponding target-specific c primer and the modified UAP-N2 primer coupled to the T7 sequence (UAP-N2_T7: TTTAATACGACTCACTATAGGGGGAAGCAGTGGTATCAACG), was used. To this end, a standard 25 μl reaction volume was applied containing 0.625 U of DreamTaq™ DNA Polymerase (Fermentas, CA, USA), 1X DreamTaq™ Buffer (Fermentas, CA, USA), 0.2 mM of dNTPs, 250 nM of each primer and 5 μl of the purified amplicon. The PCR program consisted of a single cycle of 3 min at 95 °C (initial denaturation) followed by 45 amplification cycles of 30 s at 95 °C (denaturation), 30 s at 50 °C (annealing) and 4 min at 72 °C (extension) and finishing by a single cycle of 10 min at 72 °C (final extension). The run was performed on an iQ™5 real-time PCR detection system (BioRad, Hemel Hempstead, UK). The PCR products were analysed by electrophoresis on a 1 % agarose gel (100 V, 400 mA, 60 min; INVITROGEN, CA, USA) and purified using USB® ExoSAP-IT® PCR Product Cleanup (Affymetrix, CA, USA), according to the manufacturer’s instructions, to be then sequenced via the T7 primer.

All sequencing reactions were performed on a Genetic Sequencer 3130XL using the Big Dye Terminator Kit v3.1 (Applied Biosystems, CA, USA). The sequences were aligned and analysed using “ClustalW2” software and “Nucleotide BLAST NCBI” software, respectively [35, 36].

### Verification of the transgene flanking regions by PCR amplification

The two different transgene flanking regions between the right border of the pCAMBIA cassette and the rice genome identified by the tNOS-F DNA walking method were verified by PCR amplification using the tNOS-F c primer combined to a primer designed on the rice chromosome II or III (Table 1). These oligonucleotide primers were initially evaluated *in silico* using the program “wprimersearch” from the software “wEMBOSS” [23]. A standard 25 μl reaction volume was applied containing 0.625 U of DreamTaq™ DNA Polymerase (Fermentas, CA, USA), 1X DreamTaq™ Buffer (Fermentas, CA, USA), 0.2 mM of dNTPs, 250 nM of each primer and 5 μl of Bt rice DNA (5 ng/μl). The PCR program consisted of a single cycle of 3 min at 95 °C (initial denaturation) followed by 35 amplification cycles of 30 s at 95 °C (denaturation), 30 s at 55 °C or 60 °C respectively for the rice chromosome III or II (annealing) and 1 min at 72 °C (extension) and finishing by a single cycle of 10 min at 72 °C (final extension). The run was performed on an iQ™5 real-time PCR detection system (BioRad, Hemel Hempstead, UK). The PCR products were analysed by electrophoresis on a 1 % agarose gel (100 V, 400 mA, 60 min; INVITROGEN, CA, USA) and purified using USB® ExoSAP-IT® PCR Product Cleanup (Affymetrix, CA, USA), according to the manufacturers’ instructions, in order to be sequenced. All sequencing reactions were performed on a Genetic Sequencer 3130XL using the Big Dye Terminator Kit v3.1 (Applied Biosystems, CA, USA). The identity of the obtained sequences was analysed by comparing to the software “Nucleotide BLAST NCBI” [36].

Concerning the verification of the 5’ transgene flanking region of MON863, the sequence obtained from the DNA walking strategy was compared to the available data published by Zhu et al., 2008 using the“ClustalW2” software [28]. The resulting alignment is provided in the Additional file 7.

## Additional files

Additional file 1:
**Sequences obtained from the 100 % Bt rice sample using the bidirectional p35S and tNOS DNA walking methods.** The number of the corresponding amplicons observed in Fig. 1a is indicated in brackets. The rice genome and the transgenic cassette are indicated respectively in small letter and capital letter. The hpt gene (underlined) is regulated by the promoter p35S (p35S; in italic) while the Cry1B gene (double underlined) is under the control of the tNOS terminator (tNOS; in bold). (DOCX 16 kb) Additional file 2:
**Sequences of aspecific amplicons obtained by DNA walking for the Bt rice 100 % and MON863-9.85 % samples.** The corresponding amplicon numbering is indicated in the Fig. 1a and 2a. (DOCX 20 kb) Additional file 3:
**Sequences, obtained by PCR, of the 3’transgene flanking regions of Bt rice on the rice chromosome II and III.** The rice genome and the transgenic cassette are indicated respectively in small letter and capital letter. (DOCX 15 kb) Additional file 4:
**Results from qPCR SYBR®Green screening on maize samples using the p35S, tNOS and t35S pCAMBIA methods as a decision support system.** The obtained positive and negative signals are represented by + and -, respectively. For each positive signal, the corresponding C_t_ and T_m_ are indicated. (DOCX 15 kb) Additional file 5:
**Sequences obtained from the GM maize MON863 event using the bidirectional p35S and tNOS DNA walking methods.** The number of the corresponding amplicons observed in Fig. 2a is indicated in brackets. The maize genome and the transgenic cassette are designated respectively in small letter and capital letter. The nptII gene (underlined) is under the control of the p35S promoter (p35S; in italic) and the tNOS terminator (tNOS; in bold). The 4-AS1 promoter (p-4AS1; dashed underlined) is followed by the Wheat major chlorophyll a/b binding protein gene (wtCAB; wave underlined) and Rice Actin Intron (rAct; dotted underlined) to regulate the Cry3Bb1 gene (double underlined). (DOCX 16 kb) Additional file 6:
**Visualisation of the obtained amplicons using the p35S and tNOS DNA walking methods applied on 100 ng of the GeMMA SU35-A food matrix.** For each method, four different DRT primer mixes (A-D) have been used. The analyzed amplicons are indicated by a numerotation going from 1 to 4. (PDF 66 kb) Additional file 7:
**Alignment of 5’transgene flanking region of MON863 coming from Zhu et al., 2008 (A) and the present DNA walking strategy (B) [** 28 **].** The maize genome and the transgenic cassette are indicated respectively in small letter and capital letter. (DOCX 13 kb)
