# Supplementary material for: Integrated DNA walking system to characterize a broad spectrum of GMOs in food/feed matrices
Source: BMC Biotechnol. 2015 Aug 14;15:76. doi: 10.1186/s12896-015-0191-3 (PMC4535744; doi:10.1186/s12896-015-0191-3)
Supplement: Additional file 1: — Sequences obtained from the 100 % Bt rice sample using the bidirectional p35S and tNOS DNA walking methods. The number of the corresponding amplicons observed in Fig. 1a is indicated in brackets. The rice genome and the transgenic cassette are indicated respectively in small letter and capital letter. The hpt gene (underlined) is regulated by the promoter p35S (p35S; in italic) while the Cry1B gene (double underlined) is under the control of the tNOS terminator (tNOS; in bold). (DOCX 16 kb) [file 12896_2015_191_MOESM1_ESM.docx]

| **>p35S (n° 6) [GenBank: KT184676]**  *GACGTGGTTGGAACGTCTTCTTTTTCCACGATGCTCCTCGTGGGTGGGGGTCCATCTTTGGGACCACTGTCGGCAGAGGCATCTTGAACGATAGCCTTTCCTTTATCGCAATGATGGCATTTGTAGGTGCCACCTTCCTTTTCTACTGTCCTTTTGATGAAGTGACAGATAGCTGGGCAATGGAATCCGAGGAGGTTTCCCGATATTACCCTTTGTTGAAAAGTCTCAATAGCCCTTTGGTCTTCTGAGACTGTATCTTTGATATTCTTGGAGTAGACGAGAGTGTCGTGCTCCACCATGTTATCACATCAATCCACTTGCTTTGAAGACGTGGTTGGAACGTCTTCTTTTTCCACGATGCTCCTCGTGGGTGGGGGTCCATCTTTGGGACCACTGTCGGCAGAGGCATCTTGAACGATAGCCTTTCCTTTATCGCAATGATGGCATTTGTAGGTGCCACCTTCCTTTTCTACTGTCCTTTTGATGAAGTGACAGATAGCTGGGCAATGGAATCCGAGGAGGTTTCCCGATATTACCCTTTGTTGAAAAGTCTCAATAGCCCTTTGGTCTTCTGAGACTGTATCTTTGATATTCTTGGAGTAGACGAGAGTGTCGTGCTCCACCAT*GTTGGCAAGCTGCTCTAGCC  **>hpt under the control of p35S (n° 30) [GenBank: KT184677]**  CCAAGCTCTGATAGAGTTGGTCAAGACCAATGCGGAGCATATACGCCCGGAGTCGTGGCGATCCTGCAAGCTCCGGATGCCTCCGCTCGAAGTAGCGCGTCTGCTGCTCCATACAAGCCAACCACGGCCTCCAGAAGAAGATGTTGGCGACCTCGTATTGGGAATCCCCGAACATCGCCTCGCTCCAGTCAATGACCGCTGTTATGCGGCCATTGTCCGTCAGGACATTGTTGGAGCCGAAATCCGCGTGCACGAGGTGCCGGACTTCGGGGCAGTCCTCGGCCCAAAGCATCAGCTCATCGAGAGCCTGCGCGACGGACGCACTGACGGTGTCGTCCATCACAGTTTGCCAGTGATACACATGGGGATCAGCAATCGCGCATATGAAATCACGCCATGTAGTGTATTGACCGATTCCTTGCGGTCCGAATGGGCCGAACCCGCTCGTCTGGCTAAGATCGGCCGCAGCGATCGCATCCATAGCCTCCGCGACCGGTTGTAGAACAGCGGGCAGTTCGG*TTTCAGGCAGGTCTTGCAACGTGACACCCTGTGAACGGCGGGAGATGCAATAGGTCAGGCTCTCGCTAAACTCCCCAATGTCAAGCACTTCCGGAATCGGGAGCGCGGCCGATGCAAAGTGCCGATAAACATAACGATCTTTGTAGAAACCATCGGCGCAGCTATTTACCCGCAGGACATATCCACGCCCTCCTACATCGAAGCTGAAAGCACGAGATTCTTCGCCCTCCGAGAGCTGCATCAGGTCGGAGACGCTGTCGAACTTTTCGATCAGAAACTTCTCGACAGACGTCGCGGTGAGTTCAGGCTTTTTCATATCTCATTGCCCCCCCGGATCTGCGAAAGCTCGAGAGAGATAGATTTGTAGAGAGAGACTGGTGATTTCAGCGTGTCCTCTCCAAATGAAATGAACTTCC*  **>3’ transgene flanking region on the rice chromosome III (n° 47) [GenBank: KT184678]**  **TCGCGCGCGGTGTCATCTATGTTACTAGATCTCTAG**AAGCTTGGCACTGGCCGTCGTTTTACAACGTCGTGACTGGGAAAACCCTGGCGTTACCCAACTTAATCGCCTTGCAGCACATCCCCCTTTCGCCAGCTGGCGTAATAGCGAAGAGGCCCGCACCGATCGCCCTTCCCAACAGTTGCGCAGCCTGAATGGCGAATGCTAGAGCAGCTTGAGCTTGGATCAGATTGTCGTTTCCCGCCTTCAGTTTAAACTATCAGTGTTTGcacaggctggattgagttatcattacaggaagaaaaaaaaaacaagaactatagtttattatagtgttgatgttgtgttgtcatacctgtttcatcactgaataagctgcgtttgcatgctctggggatgctcctgcagctgttcctgtggagcgtcatctccaggtggtggcgatggccattatcgccgtcgacgatcggcggcgtgcggcggccgtttctgcctccttgttcagctccggcggagttctccgctctcccgaatccatggctcccgacgaaatgcggcgacgccttctcgcagccggacggatcaaagatgagcacatcgaaggaggaggagacgccgctgtacctgaagagcaggcagtctccttcctcgatgccgttgccatccacgaactctttccagccaggctgaagaactagttcaccggcgtcgctgttggctactccaatgctccaggtttcgccgctaggagatctcagattcacttcctctgagatgtgcccgttgaaattgtttgcaaatctagcaggcacggtctgcagcaatagcaggttgactgattcag  **>3’ transgene flanking region on the rice chromosome II (n° 51) [GenBank: KT184679]**  **TCGCGCGCGGTGTCATCTATGTTACTAGATCTCTAG**AAGCTTGGCACTGGCCGTCGTTTTACAACGTCGTGACTGGGAAAACCCTGGCGTTACCCAACTTAATCGCCTTGCAGCACATCCCCCTTTCGCCAGCTGGCGTAATAGCGAAGAGGCCCGCACCGATCGCCCTTCCCAACAGTTGCGCAGCCTGAATGGCGAATGCTAGAGCAGCTTGAGCTTGGATCAGATTGTCGTTTCCCGCCTTCAGTTTAAACTATCAGTGTTTGACgccaccgccactctctcctctcctcctctctctctctctctcgcaccaccgctctcttccgccgctgcggctcacggctacgcagctctcttcccctcctcctcggctccgctctcttcgatcgatctagggtttggtcttctgttgggggattgttgttgctcttccgcgcgatcgatcgacgccgcgtcctgagggtttgaggggtttccgccctcccgccgcacgcccgcacccccgcgatgtccggccggagctcgccgatgtacgaggggctcgcgtcgcgtcccgacgagtgggacgtcgtcctcaaggtgagatgatcgtctatttttcaggattgagctgtggttttgttttgtgattagggggaggggatggcggagggggaaggggaaggggaaagggga  **>Cry1B under the control of tNOS (n° 72) [GenBank: KT184680]**  CAACCGTGAGGTTCAACTTCACCAACCCACAGAACATCTCCGATCGCGGCACTGCGAACTACTCCCAACCATACGAGTCCCCAGGCCTCCAGTTGAAGGACTCCGAGACTGAGTTGCCACCAGAGACTACTGAGCGTCCAAACTACGAGTCTTACAGCCACAGGTTGTCCCACATCGGCATCATCCTGCAGTCTAGGGTGAACGTGCCAGTGTACTCTTGGACTCACAGGTCTGCTGACCGCACTAACACCATCGGCCCAAACAGGATCACCCAAATCCCAATGGTGAAGGCCTCCGAGCTGCCACAAGGCACCACCGTGGTGAGGGGACCAGGCTTCACTGGCGGCGACATCCTCAGGAGGACCAACACTGGCGGCTTCGGTCCCATCAGGGTCACCGTCAACGGCCCATTGACCCAAAGATACCGCATCGGCTTCCGCTACGCTTCCACCGTGGACTTCGACTTCTTCGTGTCCCGTGGTGGCACCACCGTAAACAACTTCAGGTTCCTCAGGACCATGAACAGCGGTGACGAGCTCAAGTACGGCAACTTCGTGAGGCGTGCCTTCACCACCCCATTCACCTTCACCCAGATCCAAGACATCATCCGCACCTCCATCCAAGGCCTCAGCGGCAACGGCGAGGTGTACATCGACAAGATCGAGATCATCCCAGTGACCGCCACCTTCGAGGCCGAGTACGACTTGGAGAGGTAATGAATTCCTGCAGCCCAATTCGGTACCCCGACCTGCAGA**GATCGTTCAAACATTTGGCAATAAAGTTTCTTAAGATTGAATCCTGTTGCCGGTCTTGCGATGATTATCATATAATTTCTGTTGAATTACGTTAAGCATGTAATAATTAACATGTAATGCATGACGTTATTTATGAGATGGGTTTT** |
| --- |
