# Supplementary material for: Integrated DNA walking system to characterize a broad spectrum of GMOs in food/feed matrices
Source: BMC Biotechnol. 2015 Aug 14;15:76. doi: 10.1186/s12896-015-0191-3 (PMC4535744; doi:10.1186/s12896-015-0191-3)
Supplement: Additional file 2: — Sequences of aspecific amplicons obtained by DNA walking for the Bt rice 100 % and MON863-9.85 % samples. The corresponding amplicon numbering is indicated in the Fig. 1a and 2a. (DOCX 20 kb) [file 12896_2015_191_MOESM2_ESM.docx]

| **>5’ transgene flanking region (n°1) [GenBank: KT184681]**  tttggcattcatctctatttgcgctcatgaaaaatagatagcctatgattcgagtagtatcgaagggtcgttcaatagcatagctcttttttctcttcgagaaagctcgcatctaatctgtaggccggacggctttgtttgccctagcttggcgaatcgcgcccctgaccgttctcgcgaagtctttgcaacggctgggaaacctgtctacgaagctaagcatattgcacgccgaccatcaaatacgagattgggccccttctcaaagatggaatggcccagcccaataaaggaaggttaacgtacgcgatgccttccatttgtacgaatcgcgaacataccacgcacgaccggacgtagagccaaaattcactggcagaccgagtcgggcgcaggtgccagatcctcaaagtatcgtaaagttaagttaagtatcgtaaagtatcgatcagcctagtgtaccaaccacgtggtacgacgggcactcaaagacctggcgaatgagggcccacccaagagcgcttatgtcatatgggaactcttggctggaaacaatccttatggtttttatatccggttagaataataagaaagaatcaaagtccaggttggttggtgagcctagtgataggagactatctagcttggttcggagagcacttgttgggtttaagattagttttttgctaaatgttacggcctaaatgctgaactattgaccctacttgttcggatgggtgttcaccccaaagtgt*ACCAAGCTTTCCGATCCTACCTGTCACTTCATCAAAAGGACAGTAGAAAAGGAAGGTGGCACCTACAAATGCCATCATTGCGATAAAGGAAAGGCTATCATTCAAGATGCCTCTGCCGACAGTGGTCCCAAAGATGGACCCCCACCCACGAGGAGCATCGTGGAAAAAGAAGACGTTCCAAC*  **>p35S regulating nptII (n° 7) [GenBank: KT184682]**  *TCATTTCATTTGGAGAGGACACGCTGAATCACCAGTCTCTCTCTACAAGATC*GGGGATCTCTAGCTAGACGATCGTTTCGCATGATTGAACAAGATGGATTGCACGCAGGTTCTCCGGCCGCTTGGGTGGAGAGGCTATTCGGCTATGACTGGGCACAACAGACAATCGGCTGCTCTGATGCCGCCGTGTTCCGGCTGTCAGCGCAGGGGCGCCCGGTTCTTTTTGTCAAGACCGACCTGTCCGGTGCCCTGAATGAACTGCAGGACGAGGCAGCGCGGCTATCGTGGCTGGCCACGACGGGCGTTCCTTGCGCAGCTGTGCTCGACGTTGTCACTGAAGCGGGAAGGGACTGGCTGCTATTGGGCGAAGTGCCGGGGCAGGATCTCCTGTCATCTCACCTTGCTCCTGCCGAGAAAGTATCCATCATGGCTGATGCAATGCGGCGGCTGCATACGCTTGATCCGGCTACCTGCCCATTCGACCACCAAGCGAAACATCGCATCGAGCGAGCACGTACTCGGATGGAAGCCGGTCTTGTCGATCAGGATGATCTGGACGAAGAGCATCAGGGGCTCGCGCCAGCCGAACTGTTCGCCAGGCTCAAGGCGCGCATGCCCGACGGCGAGGATCTCGTCGTGACCCATGGCGATGCCTGCTTGCCGAATATCATGGTGGAAAATGGCCGCTTTTCTGGATTCATCGACTGTGGCCGGCTGGGTGTGGCGGACCGCTATCAGGACATAGCGTTGGCTACCCGTGATATTGCTGAAGAGCTTGGCGGCGAATGGGCTGACCGCTTCCTCGTGCTTTACGGTATCGCCGCTCCCGATTCGCAGCGCATCGCCTTCTATCGCCTTCTTGACGAGTTCTTCTGAGCGGGACTCTGG  **>p4AS-1, followed by wtcab and rAct, regulating Cry3Bb1 (n°6) [GenBank: KT184683]**  TCATTTCATTTGGAGAGGACACGCTGACAAGCTAGCTTGGCTGCAGGTAGATCCTAGAACCATCTTCCACACACTCAAGCCACACTATTGGAGAACACACAGGGACAACACACCATAAGATCCAAGGGAGGCCTCCGCCGCCGCCGGTAACCACCCCGCCCCTCTCCTCTTTCTTTCTCCGTTTTTTTTTCCGTCTCGGTCTCGATCTTTGGCCTTGGTAGTTTGGGTGGGCGAGAGGCGGCTTCGTGCGCGCCCAGATCGGTGCGCGGGAGGGGCGGGATCTCGCGGCTGGGGCTCTCGCCGGCGTGGATCCGGCCCGGATCTCGCGGGGAATGGGGCTCTCGGATGTAGATCTGCGATCCGCCGTTGTTGGGGGAGATGATGGGGGGTTTAAAATTTCCGCCGTGCTAAACAAGATCAGGAAGAGGGGAAAAGGGCACTATGGTTTATATTTTTATATATTTCTGCTGCTTCGTCAGGCTTAGATGTGCTAGATCTTTCTTTCTTCTTTTTGTGGGTAGAATTTGAATCCCTCAGCATTGTTCATCGGTAGTTTTTCTTTTCATGATTTGTGACAAATGCAGCCTCGTGCGGAGCTTTTTTGTAGGTAGAAGTGATCAACCATGGCCAACCCCAACAATCGCTCCGAGCACGACACGATCAAGGTCACCCCCAACTCCGAGCTCCAGACCAACCACAACCAGTACCCGCTGGCCGACAACCCCAACTCCACCCTGGAAGAGCTGAACTACAAGGAGTTCCTGCGCATGACCGAGGACTCCTCCACGGAGGTCCTGGACAACTCCACCGTCAAGGACGCCGTCGGGACCGGCATCTCCGTCGTTGGGCAGATCCTGGGCGTCGTTGGCGTCCCCTTCGCAGGTGCTCTCACCTCCTTCTACCAGTCCTTCCTGAACA  **>tNOS followed by p4AS-1 coupled to wtcab and rAct to regulate Cry3Bb1 (n°10) [GenBank: KT184684]**  **CGCGCGGTGTCATCTATGTTACTAGATCGGG**GATATCCCCGCGGCCGCGTTAACAAGCTTCTGACGTAAGGGATGACGCACCTGACGTAAGGGATGACGCACCTGACGTAAGGGATGACGCACCTGACGTAAGGGATGACGCACTCGAGATCCCCATCTCCACTGACGTAAGGGATGACGCACAATCCCACTATCCTTCGCAAGACCCTTCCTCTATATAAGGAAGTTCATTTCATTTGGAGAGGACACGCTGACAAGCTAGCTTGGCTGCAGGTAGATCCTAGAACCATCTTCCACACACTCAAGCCACACTATTGGAGAACACACAGGGACAACACACCATAAGATCCAAGGGAGGCCTCCGCCGCCGCCGGTAACCACCCCGCCCCTCTCCTCTTTCTTTCTCCGTTTTTTTTTCCGTCTCGGTCTCGATCTTTGGCCTTGGTAGTTTGGGTGGGCGAGAGGCGGCTTCGTGCGCGCCCAGATCGGTGCGCGGGAGGGGCGGGATCTCGCGGCTGGGGCTCTCGCCGGCGTGGATCCGGCCCGGATCTCGCGGGGAATGGGGCTCTCGGATGTAGATCTGCGATCCGCCGTTGTTGGGGGAGATGATGGGGGGTTTAAAATTTCCGCCGTGCTAAACAAGATCAGGAAGAGGGGAAAAGGGCACTATGGTTTATATTTTTATATATTTCTGCTGCTTCGTCAGGCTTAGATGTGCTAGATCTTTCTTTCTTCTTTTTGTGGGTAGAATTTGAATCCCTCAGCATTGTTCATCGGTAGTTTTTCTTTTCATGATTTGTGACAAATGCAGCCTCGTGCGGAGCTTTTTTGTAGGTAGAAGTGATCAACCATGGCCAACCCCAACAATCGCTCCGAGCACGACACGATCAAGGTCACCCCCAACTCCGAGCTCCAGACCAACCACAACCAGTACCCGCTGGCCGACAACCCCAACTCCACCCTGGAAGAGCTGAACTACAAGGAGTTCCTGCGCATGACCGAGGACTCCTCCACGGAG  **>nptII followed by tNOS (n°16) [GenBank: KT184685]**  GGGTGTGGCGGACCGCTATCAGGACATAGCGTTGGCTACCCGTGATATTGCTGAAGAGCTTGGCGGCGAATGGGCTGACCGCTTCCTCGTGCTTTACGGTATCGCCGCTCCCGATTCGCAGCGCATCGCCTTCTATCGCCTTCTTGACGAGTTCTTCTGAGCGGGACTCTGGGGTTCGAAATGACCGACCAAGCGACGCCCAACCTGCCATCACGAGATTTCGATTCCACCGCCGCCTTCTATGAAAGGTTGGGCTTCGGAATCGTTTTCCGGGACGCCGGCTGGATGATCCTCCAGCGCGGGGATCTCATGCTGGAGTTCTTCGCCCACCCCCCGGATCCCCATGGGAATTCCC**GATCGTTCAAACATTTGGCAATAAAGTTTCTTAAGATTGAATCCTGTTGCCGGTCTTGCGATGATTATCATATAATTTCTGTTGAATTACGTTAAGCATGTAATAATTAACATGTAATGCATGACGTTATTTATGAGATGG** |
| --- |
