# Supplementary figures and images for: Integrated DNA walking system to characterize a broad spectrum of GMOs in food/feed matrices
Source: BMC Biotechnol. 2015 Aug 14;15:76. doi: 10.1186/s12896-015-0191-3 (PMC4535744; doi:10.1186/s12896-015-0191-3)

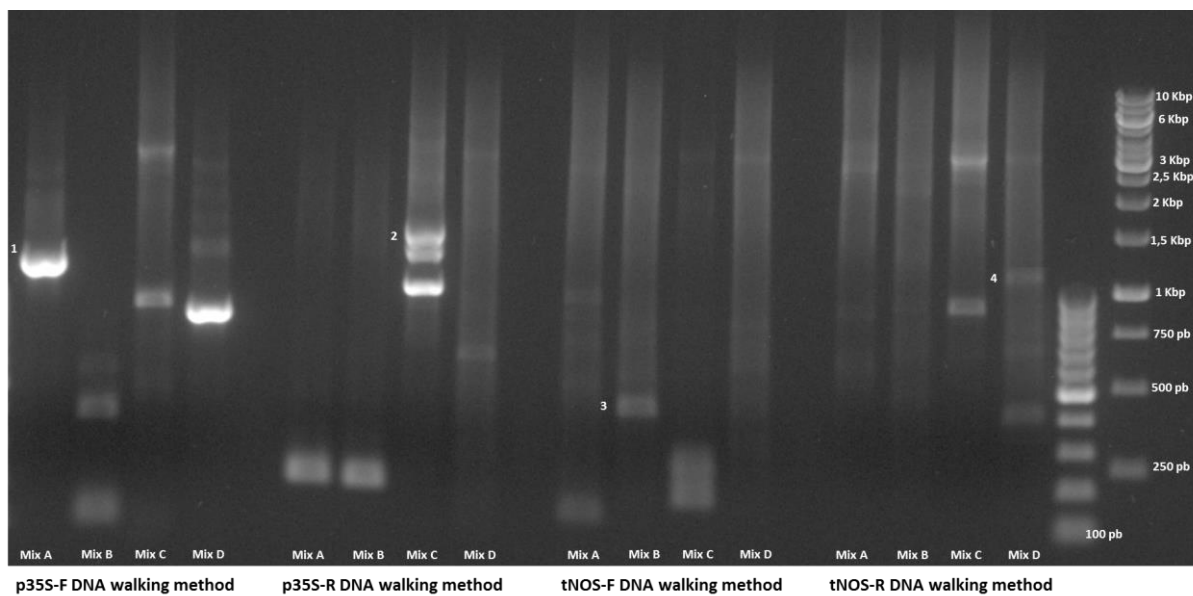

Supplement: Additional file 3: — Sequences, obtained by PCR, of the 3’transgene flanking regions of Bt rice on the rice chromosome II and III. The rice genome and the transgenic cassette are indicated respectively in small letter and capital letter. (DOCX 15 kb) [file 12896_2015_191_MOESM3_ESM.docx]
