# Supplementary material for: Integrated DNA walking system to characterize a broad spectrum of GMOs in food/feed matrices
Source: BMC Biotechnol. 2015 Aug 14;15:76. doi: 10.1186/s12896-015-0191-3 (PMC4535744; doi:10.1186/s12896-015-0191-3)
Supplement: Additional file 4: — Results from qPCR SYBR®Green screening on maize samples using the p35S, tNOS and t35S pCAMBIA methods as a decision support system. The obtained positive and negative signals are represented by + and -, respectively. For each positive signal, the corresponding Ct and Tm are indicated. (DOCX 15 kb) [file 12896_2015_191_MOESM4_ESM.docx]

| **Sample** | **Amplicon** | **NCBI Reference Identifier** | **Sequence** |
| --- | --- | --- | --- |
| **Bt rice 100%** | n°19 | AP003846.2 | AGAAATCCATGGCCACCTCCTTGGTCAGCTGGAGTGACTAGAAGGTGGAGGTGTGGCATGTACCTTGGTCCGTATTCAATTTCTTTTGGAAAAGAGTATATTTTTTGAAAGAAATTTGAAAAGAGTATATTGGAAACCACCATGGCCTCCACCTAATAGGAATGATATTATGCACCTCCTGACTAATTCACACACATTTGTAAATTAGAGAAGGCAAATTTGGCTAAATTTAAAGCAAGGGAATGTGAAATATTATTA |
|  | n°20, 21, 61, 88, 92 and 94 | BA000029.3 | AGACCTTCCAGCTTGATCTTATTGATGGATCATTCACTCAATCAAAACATTGACGCACGCGCCTTCTTTCTAGGTAATTGAATAGAAAAGTGACGGTCTCATCAGCGGCCTGGGAGTGAATGTGGAAGCTTAACACTAGCAACGGGCAGAACAGTTGGTAAAGGAGATCATTGGCACTATGGCGCATGTATGTTGAGTTC |
|  | n°22 | NM_001055107.1 | GCAAACCCCGGTCCGATGAAGTCAGAGAGGATGGAGAGAATCCAGCTGAGACCATCTCATTTAGCAGATACTTTGCCTTCTCCACTGCCCCAGTCCCAAAGAGACCAGCAACCAGTGTGTTGTATGTGATGAGGTTAGGCTTTATGGAGCTCATCTTCATTTCATGAAGGAGTTTTAGAGCTTTAGCAGTCTCTCCTTTCCTGCAATGCGAAACAATCATAGTGTTATATGTTGATTGATCAGGTTTTAATCCCATATTTCTCATCTCTGTCAAGAAGGATTTTGCTTCCTTAAACTTCCCAAGCATGCAAAGGCAATTGATAAACACATTATAAACAACTGCATCGGGCAACATGTTTCTGTCCATTAACTCCTGACCAAACTTAAAAGCAGTAGGCATGTCGCCTGCTTTGAAGAGCCCATCTATTAAAGTGGTATAGTTTACGTGATCTAGTGACAGGCCACTC |
|  | n°23 | AC099040.10 | TTTAAAAACTTGATTATTCTTTCGTGTTTCTGTCTTCTGCATCTTTTTTACTGTTCATTTTTCTCCTAGTACCTTTGTAGACTGGTGTCTACATTAGATTGTATTTTGAAGATCAATGTGTAGGGACAGGACACATTGATCTTCAAAATACAATCTAATGTAGACACCAGTCTACAAAGGTACTAGGAGAAAAACGAACAGTA |
|  | n°36 | AP003196.3 | ATTTTTTTTTTAATAAAAATTGAAGATGTTTTTGCCGGTACTTTGGTAGGTCATCCGTGTATGAGTCGATTTTTAAGTTCGTTTGCTTTTGGAAATACATATCCGTATTTGAGTCTGAGTCTTTTTAAGTTCATTCGTTTTTGGAAATACATAATGGATAGCTTTAAAAATATTGCATGCTAATTTTAGACGATCAGACTCTTAACTGCAGCTCATGATTTTCTAAAAAACATATATCTAAGCGAATTCCTACAGTATTGATGTACTGCATACAGGCCATATAAAAGA |
|  | n°37 | AL831795.4 | CCACTTGATCTGGAGGGTTGATTGTCCCCCCATTGGATGTCATGAGGCGCATGCCACCGGTCCTCACTGGCACTTGCCCAGGTTGAGACTCCGATGCTATGGCGTGGAGCTTGGGTCCATCCGGGTGCATCCCCTGTTGGTGCCCATCCTGCCTCATACATCTGTACGGGTGTTGAAGGATAGTGAA |
|  | n°38 | AP004134.3 | TGGGCACCAACTGGGGATGCACGCGGATGGACCCAAGCTCCACGCCATAGCATCGGAGTCTCAACCTGGGCAAGTGCCAGTGAGGACCGGTGGCATGCGCCTCATGGCATCCACTGGGGGGGAACAATCAACCCTCCCAGATCAAGTGGTGTGCC |
|  | n°39 | AC068654.2 | ATAATGATTAAGAGAAATGACGATGGGTGTGCCGTTAAGCAACATGGTCACGACGGTTGGCGGTACTTATGAAGAGTAAAAATAAAACCAATTGATAATCATGCTCGATTTTTAAAATCTCAATGAC |
|  | n°40 | AP004592.3 | CCCTGCATCCTCCCTTCCATCTCCACACCTGCAAAGACTGTCCCCAAGAACCCCCCACAAGCTCACCAAAGCAGCCTCATCGAACTCGCAAAACCCCAAATCTTCAGCGTCCCAAAGCCAAAACCCAGGTCACGAGAAAACCTCAAATATTCCGAGATTTGACGAAATGGGCACCCACCCCACCACCTGCAAGATCAAGAATCAGGCGGCTCTCACGGATCCCAGCAAGAGAGGAGATGCATCGAACGAGTCGGCGTGAGACGGCGAGGCCTACCTG |
|  | n°41 | AC114896.5 | CTCCGAAGTGAGCTCCATGTCATCATGGGGTACATCAACACCTGTACAATCAACTTTCCAAATACTTGGATGCAAAGAGATTAGATGCAACTTCATCTTGTGCCTCCAAGCGGCGTAATGTGTCCCATCAGATTGAGGAGCACGCCCGGAAGGCACGGAGACGAAGTTATGGGGGGGAATGGTAAGTTTGCTGTAATCAAAAGGTATAGTAGCTCCCTTGGGTGGTGGAGTTCCCTTGCTGCTCGCTCCATCTTCATTTCCATTTTCCCCGTTCTTTCCCCCTAAGTCCGACGTCGTGGATCGCTCAAAGCGGTGAAGCTAGAATCAAGAGCACAAGGCTCTGATACAAATTGAACGCGTTGATGAAGCCTAGAGGGGGGGTGAATAGACTGTCCCTGAAAACTTAAAAACAAATCGCAGCGATAAAATTCAAATAGACCCGGAACTTCCTGGTAAGGAACTCCGGAACTTCCGGCCTGCCAGGCCCGGAACTTCCGGTGTTCTAGGATAGGAACTTCCGGGTAACAGAACAGAGATGAAATTCAAATTTTGAAACATGAGACTAAGCCAATCTTCACAAGAAGGTGCACAGGTATTCTAAGCAGAGGATAGATCACAGAATCATCCACAGAAACATCACAGAAAAAGACAAGAGCGATTTTTTCCCGAAGTTCGGATCTTGCGA |
|  | n°42 | AP004558.3 | CCCCCGGCGCTTCTTCTCCAATCCACCCCCCTCTAGCGCTTCTCCTCCCCCTCCCCCCCCAATCTTTGTTTCATCCGAGCTTTCACTTCTGCGAGGTTGTGTGTTCTTCTACGGGATTCTCTCCCCGGCGATTCTCTGGCCGCTTCTCCTCCCTCCGTATATCTATTGAGTC |
|  | n°60 | AP003616.4 | TATTGCTTTAAACTTGTGTTCGGGCAAAATGACGTATGCATTAACACATTATTAATTAAATTTTAGCTAATTTTTTAAAAATAGATTAATATGATTTTTTTTAAAGCAACTTTTGTATAGATTTTTTTTAAAAAATACACCGTTTACTCGTTTGAAAAACGTAAATGTGTAAAACAAGAGATGAGTTAGGAAAGTGAAAAAAAAAACTCAGCTGAGTCATTTATCCAAAATCCGTTGCACCACACGTGAACATTAGGCTGCCCCTAGATCCCCAGCTGTAATAATATCCTAAGAAATATTT |
|  | n°62-63 | AP005091.3 | AATTGTTTGCTAGATATGCTATAGAGGAGCAAAGTGTCATTTACCTCCTTACCCTCATCATTAGCAGTATCATCATCATTATCATTACTACTTGCATCGGTTTCATCATCATCAGTTTCCTCCATAGACTCTTCATCGTCACTTGCCTCAGTAAACTCATCTTCGTCGCTCACATCCTCGTCATCACCACTTTCTTCATTGTCCTGGTCGTCGTCGCTTTCCTCTAGAGACATATCATAGTTGTAATGAAACAATAACATCGGAGCTAATTTATGATCCAACGAAG |
|  | n°64 | AP005199.3 | TTGCTACTGCTTGCCTTTTCTGACTCGCTCTCTCTCTCTCTCTCTATATATATATATATATATATATATATGGAGTATATAGCTTTTTTTTCCTACTAGTGAGAGAGAGAGAGAGAAGCCATTATTATTTTTCCCGGAAGAACAAGAGGAAACGAATAAAAAGGAAATCTACATCAATAAAGCTAATTCACAGTTCCTTGTGCTATACAAACGTACAGTAGATTATTGATTAGCAACAGATTCTCACTGGATTATATCGTGAAAATTAG |
|  | n°65 and 90 | AC130607.2 | TTGCTAATTACCGTGTACGCGCAATCAACTCAAGTCGCGAGCGTTTCTGCATGTCCTCGCTCGTGGCCCACAGCCCTGCGTGCAGCCCATCGATATCATCTTTTTGCTTCTTTTTTGCGCAACGTTTCTTTTTCTCTTTTTTTTTTCACGCGAAATGCCGCACGTGTGCATGGCGTGGAGAAAAAACCGTCAATTTTGTTTCCAAAAATCACGACCTATTAAGATGTCCTTCTTGTTTTCACAAACATTGTTTTCTGTTTTGAATATATTATAATAATAATAATAATAATAATAAAAC |
|  | n°89 and 91 | AP004258.3 | AAATGCTCTTGCGGATCTTTGAATTTTATACGTGAATTCAGAAAAAAGTTAAAAGAAATAACATAACGTAAATAGGAACCTGTAATAATTAAACAGAGTAGAATTGCTGGATCTATATCATTGCGGGCAAATCAAGTGGCTTATATGTTTGGGAGTCAACAACAAGGGTCCAGCTAGACTGGTAGTCTAGAGCGCATATCCGTAGTGTGTGTGACGCGACTATGTAAACCCAACAAACCATGGACGTCGATGACGACGAGGTGGAACGACTG |
|  | n°93 | AC083945.3 | CCAACCGTCGCCTTCTGGGTTGCCTGCTTGGTTTTCCAGCCAGCGCCATGGATTCACGCTCCTCCGTCAAACGGTGAGTCCTCCATGCGCCTCTGGTTCGAGGAATCTAGTTTTTATACTTCATTTTCCGAACGACGAATCTATCTCTGCTGCTTCTCAATCGCATCCACCAGAGCCTCAATCTACTGCTGCTGCTGTGCCTCGCTCACAATGGAT |
| **MON863-9.85% CRM** | n°3, 14 and 15 | AC196084.4 | ATTCTCACGAATATTGTTTACGAGATGAGGATCAAGAACGGTAAAGGACCTTAGCATTAGGCGGACGACGTCGTGGTCCGTCCATCGCGTGCTTCCGTAGCTCCTTATTTTGTTGATAAGGGTCTTGAGCCGGTTGTAAGTTTGGGTTGGCTCCTCTCCCCTTATCATTGCAAATCGTCCAAGCTCGCCCTCCACCAACTCCATTTTAGTGAGCATGGTGATGTCGTTCCCCTCGTGAGAGATCTTGAGGGTGTCCCATATCTGCTTGGCGTTGTCCAAGCCGCTCACCTTATTGTACTCTTCCCTGCACAAAGATGCTAATAGAACAGTAGTAGCTTGTGCATTTTTATGGATTTGTTCATTTATAAGCATAGGGCTATCTGAGCTATCAAATTTCATTCCATTCTCTACAATCTCCCATATGCTTGGATGGAGAGAGAATAAGTGACTACGCATTTTGTGACTCCAAAATCCGTAGTCTTCCCCATCGAAGTGTGGAGGTTTGCCAAGAGGAATGGAAAGCAAATGCGAATTCGAACTATGTGGAATACGAGAATAATCAAATGAAAAGTTCGAATTGACCGTCTTCCTGTAGTCGTTGTCGTCGTCCTTTTGGGAAGAAGTAGACTCATCGCTATCGTCGTAGTAGACGATCTCCTTGATGCGCCTTGTCTTCTTCTTCTTCTCATCTTTACGCTTGTGGCCCAAGCCCGAGTCAGTAGGCTTGTCATCCTTCGGCTCGTTGACGAAGGACTCCTTCTCCTTGTCGTTGA |
