# Supplementary material for: Integrated DNA walking system to characterize a broad spectrum of GMOs in food/feed matrices
Source: BMC Biotechnol. 2015 Aug 14;15:76. doi: 10.1186/s12896-015-0191-3 (PMC4535744; doi:10.1186/s12896-015-0191-3)
Supplement: Additional file 5: — Sequences obtained from the GM maize MON863 event using the bidirectional p35S and tNOS DNA walking methods. The number of the corresponding amplicons observed in Fig. 2a is indicated in brackets. The maize genome and the transgenic cassette are designated respectively in small letter and capital letter. The nptII gene (underlined) is under the control of the p35S promoter (p35S; in italic) and the tNOS terminator (tNOS; in bold). The 4-AS1 promoter (p-4AS1; dashed underlined) is followed by the Wheat major chlorophyll a/b binding protein gene (wtCAB; wave underlined) and Rice Actin Intron (rAct; dotted underlined) to regulate the Cry3Bb1 gene (double underlined). (DOCX 16 kb) [file 12896_2015_191_MOESM5_ESM.docx]

**>3’ transgene flanking region on the rice chromosome II**

TCGCGCGCGGTGTCATCTATGTTACTAGATCTCTAGAAGCTTGGCACTGGCCGTCGTTTTACAACGTCGTGACTGGGAAAACCCTGGCGTTACCCAACTTAATCGCCTTGCAGCACATCCCCCTTTCGCCAGCTGGCGTAATAGCGAAGAGGCCCGCACCGATCGCCCTTCCCAACAGTTGCGCAGCCTGAATGGCGAATGCTAGAGCAGCTTGAGCTTGGATCAGATTGTCGTTTCCCGCCTTCAGTTTAAACTATCAGTGTTTGAcgccaccgccactctctcctctcctcctctctctctctctctcgcaccaccgctctcttccgccgctgcggctcacggctacgcagctctcttcccctcctcctcggctccgctctcttcgatcgatctagggtttggtcttctgttgggggattgttgttgctcttccgcgcgatcgatcgacgccgcgtcctgagggtttgaggggtttccgccctcccgccgcacgcccgcacccccgcgatgtccggccggagctcgccgatgtacgaggggctcgcgtcgcgtcccgacgagtgggacgtcgtcctcaaggtgagatgatcgtctatttttcaggattgagctgtggttttgttttgtgattagggggaggggatggcggagggggaaggggaaggggaaaggggaatcgctcctggtgggggtttgggggaagcgtggggggcttgcgtacttatcgttttcgtagggtttgattgacgaaatgtgtggtgttgcgggatttgtgtacgctag

**>3’ transgene flanking region on the rice chromosome III**

TCGCGCGCGGTGTCATCTATGTTACTAGATCTCTAGAAGCTTGGCACTGGCCGTCGTTTTACAACGTCGTGACTGGGAAAACCCTGGCGTTACCCAACTTAATCGCCTTGCAGCACATCCCCCTTTCGCCAGCTGGCGTAATAGCGAAGAGGCCCGCACCGATCGCCCTTCCCAACAGTTGCGCAGCCTGAATGGCGAATGCTAGAGCAGCTTGAGCTTGGATCAGATTGTCGTTTCCCGCCTTCAGTTTAAACTATCAGTGTTTGcacaggctggattgagttatcattacaggaagaaaaaaaaaacaagaactatagtttattatagtgttgatgttgtgttgtcatacctgtttcatcactgaataagctgcgtttgcatgctctggggatgctcctgcagctgttcctgtggagcgtcatctccaggtggtggcgatggccattatcgccgtcgacgatcggcggcgtgcggcggccgtttctgcctccttgttcagctccggcggagttctccgctctcccgaatccatggctcccgacgaaatgcggcgacgccttctcgcagccggacggatcaaagatgagcacatcgaaggaggaggagacgccgctgtacctgaagagcaggcagtctccttcctcgatgccgttgccatccacgaactctttccagccaggctgaagaactagttcaccggcgtcgctgttggctactccaatgctccaggtttcgccgctaggagatctcagattcacttcctctgagatgtgcccgttgaaattgtttgcaaatctagcaggcacggtctgcagcaatagcaggttgactgattcaggtttttttttttcatacttcagttctttctgaggtacacagtgatatgaatagagagattcgtgcatgaatttactttacttcaaaaacaaaaagcgcttgagtttaccatggaaagacatgcaaaatgaaatcttagagagagagagagagagagagagagtaagattttgcatttagccttatatggtctcatggcctgatggcccctcaaaaaggctggaaaaggcttgagtacct
