# Supplementary material for: Integrated DNA walking system to characterize a broad spectrum of GMOs in food/feed matrices
Source: BMC Biotechnol. 2015 Aug 14;15:76. doi: 10.1186/s12896-015-0191-3 (PMC4535744; doi:10.1186/s12896-015-0191-3)
Supplement: Additional file 6: — Visualisation of the obtained amplicons using the p35S and tNOS DNA walking methods applied on 100 ng of the GeMMA SU35-A food matrix. For each method, four different DRT primer mixes (A-D) have been used. The analyzed amplicons are indicated by a numerotation going from 1 to 4. (PDF 66 kb) [file 12896_2015_191_MOESM6_ESM.pdf]

|  | **Screening markers** | | |
| --- | --- | --- | --- |
| **Samples** | **p35S** | **tNOS** | **t35S pCAMBIA** |
| **MON863 9.85 %** | **+**  **(C_t_: 25.5; T_m_: 76C)** | **+**  **(C_t_: 25.2; T_m_: 72°C)** | **-** |
| **Food matrix (GeM SU34-B)** | **+**  **(C_t_: 29.7; T_m_: 76°C)** | **+**  **(C_t_:29.3; T_m_:72°C)** | **-** |
