# Supplementary material for: Integrated DNA walking system to characterize a broad spectrum of GMOs in food/feed matrices
Source: BMC Biotechnol. 2015 Aug 14;15:76. doi: 10.1186/s12896-015-0191-3 (PMC4535744; doi:10.1186/s12896-015-0191-3)
Supplement: Additional file 7: — Alignment of 5’transgene flanking region of MON863 coming from Zhu et al., 2008 (A) and the present DNA walking strategy (B) [ 28 ]. The maize genome and the transgenic cassette are indicated respectively in small letter and capital letter. (DOCX 13 kb) [file 12896_2015_191_MOESM7_ESM.docx]

A: sequence of 5’ transgene flanking region of MON863 from Zhu et al., 2008

B: sequence of 5’ transgene flanking region of MON863 from DNA walking strategy

CLUSTAL O(1.2.1) multiple sequence alignment

A agggccggacggctttgtttgccctagcttggcgaatcgcgcccctgaccgttctcgcga

B ag-gccggacggctttgtttgccctagcttggcgaatcgcgcccctgaccgttctcgcga

** *********************************************************

A agtctttgcaacggctgggaaacctgtctacgaagctaagcatattgccacgccgaccat

B agtctttgcaacggctgggaaacctgtctacgaagctaagcatattgc-acgccgaccat

************************************************ ***********

A caaatacgagattgggccccttctcaaagatggaatggcccagcccaataaaggaaggtt

B caaatacgagattgggccccttctcaaagatggaatggcccagcccaataaaggaaggtt

************************************************************

A aacgtacgcgatgccttccatttgtacgaatcgcgaacataccacgcacgaccggacgta

B aacgtacgcgatgccttccatttgtacgaatcgcgaacataccacgcacgaccggacgta

************************************************************

A gagccaaaattcactggcagaccgagtcgggcgcaggtgccagatcctcaaagtatcgta

B gagccaaaattcactggcagaccgagtcgggcgcaggtgccagatcctcaaagtatcgta

************************************************************

A aagttaagttaagtatcgtaaagtatcgatcagcctagtgtaccaaccacgtggtacgac

B aagttaagttaagtatcgtaaagtatcgatcagcctagtgtaccaaccacgtggtacgac

************************************************************

A gggcactcaaagacctggcgaatgagggcccacccaagagcgcttatgtcatatgggaac

B gggcactcaaagacctggcgaatgagggcccacccaagagcgcttatgtcatatgggaac

************************************************************

A tcttgactggaaacaatccttatggtttttatatccggttagaataataagaaagaatca

B tcttggctggaaacaatccttatggtttttatatccggttagaataataagaaagaatca

***** ******************************************************

A aagtccaggttggttggtgagcctagtgataggagactatctagcttggttcggagagca

B aagtccaggttggttggtgagcctagtgataggagactatctagcttggttcggagagca

************************************************************

A cttgttgggtttaagattagttttttgctaaatgttacggcctaaatgctgaactattga

B cttgttgggtttaagattagttttttgctaaatgttacggcctaaatgctgaactattga

************************************************************

A ccctacttgttcggatgggtgttcaccccaaagtgtACCAAGCTTTCCGATCCTACCTGT

B ccctacttgttcggatgggtgttcaccccaaagtgtACCAAGCTTTCCGATCCTACCTGT

************************************************************

A CACTTCATCAAAAGGACAGTAGAAAAGGAAGGTGGCACCTACAAATGCCATCATTGCGAT

B CACTTCATCAAAAGGACAGTAGAAAAGGAAGGTGGCACCTACAAATGCCATCATTGCGAT

************************************************************

A AAAGGAAAGGAATCGTCGACCTGCAGGCATGCAAGCTTGGCAC

B AAAGGAAAGGCTATCATTCAAGATGCCTCTGCCGACAGTGGTC

*******************************************
